# Methods of Anesthesia, Old and New

**Published:** 1907-03

**Authors:** W. F. Waugh

**Affiliations:** Chicago


**For Texas Medical Journal.**

### Methods of Anesthesia, Old and New.

BY W. F. WAUGH, M. D., CHICAGO.

The elder Sothern, as Lord Dundreary, gave us the expression: "One of the things no fellow can find out." The latest exemplification of this matter may be found in recent statements concerning anesthesia. On one side we are confronted with the assertion that anesthesia by ether inhalation has been so perfected that it occasioned but one death out of over 15,000 administrations. The statistics showing by whom and under what circumstances this remarkable result has been achieved are judiciously suppressed.

But here comes our difficulty: Scarcely a journal do we pick up which does not contain a description of a new inhaler for ether, or of a new and extraordinary difficult and complicated method of administering this safe anesthetic, or of the perils encountered by the patients to whom it is administered, and the means of obviating them. Why under heaven, if ether administered by former methods is so much less fatal than a hearty meal, should we desert the methods which have produced such results, and cocainize the throat, pack it with gauze, introduce the ether vapor through a tube, pass it through the nostrils and into the larynx, with a whole lot more technique too tedious for description here? Somehow these things do not seem to harmonize.

In addition to all this, we note in the *Medical Standard* for March an excellent editorial presenting a plea for expert anesthetics. The importance and the difficulties of administering anesthetics are well described, and the writer in making his plea does so because he himself had a misfortune to lose a child on the operating table. This was a second operation, and at the first one the boy came very near dying from an error of the anesthetist. An expert was therefore secured for the second operation, but the boy died nevertheless. The following significant expression is made in this editorial: "What is the correct status of anesthesia mortality is hard to determine."

Nevertheless, we are called upon to believe that ether anesthesia is so perfected that but one death is occasioned in more than 15,000 administrations. As this statement is given as a reason for the general preference of ether by the profession as a whole, it is evidently intended to convey the impression that these results are to be obtained by the general profession as a whole, and are not simply of specially skilled administration, by specially expert administrators, in selected cases, in specially designed hospitals. Let us know, however, whether equal results are to be expected when the surgeon operates under the stress of emergency, in the patient's home, or under such conditions as accident cases present, without any professional assistance whatsoever. These are the conditions under which hyoscine-morphine and cactin anesthesia is winning laurels. Let justice prevail, and make a fair comparison with ether under similar conditions.

*The Medical Times,* in discussing anesthesia, remarks, that despite the most anxious care, fatalities do occur under anesthesia. In some of these, as in the case of Colonel Shepard, who partook of a solid breakfast the morning before operation, some decent excuse may be found for attributing the fatality to something else than the ether employed. That is all right, and we only ask that the same consideration be extended to the new hypodermic anesthetic—"hyoscine, morphine and cactin compound."

In a recent issue of the *Journal of the A. M. A.,* Dr. H. C. Wood (not the real Wood of *Wood's Therapeutics,* but a son of his), comes out, in answer to a correspondent who wants to know something about the value of hyoscine-morphine anesthesia, and with bitter vindictiveness condemns the method, exposing, as he goes along, his ignorance of the subject under discussion. Wood insists upon quoting the failures which have followed the use of commercial scopolamine and morphine, evidently having had no experience with "hyoscine-morphine and cactin compound" (Abbott), probably not with either, and states that this drug and hyoscine are one and the same thing—which is well known not to be the case. He takes the opportunity to call Abbott (who, among other excellent work for the profession, has lately called attention to the immense value of pure hyoscine with morphine and cactin as an anesthetic in major surgery and obstetrics) a "falsifier." In his eagerness to vent his animosity Wood ignores the fact that Abbott in his articles recommends *atroscine-free hyoscine*, morphine and cactin in definite proportions, decrying the use of commercial scopolamine and morphine, and deluges the correspondent (who ventured to ask about the efficacy of the Abbott formula) with gruesome data regarding the fatalities which have marked the use of scopolamine and morphine alone, the bad symptoms produced by scopolamine being due, according to best authority, to the atroscine present, which acts like atropine which should never be used, it being, according to Thrush and many others, directly antagonistic to both hyoscine and morphine.

The great difference in the physiological action of scopolamine and atroscine-free hyoscine is not for one moment considered by Wood (probably because it is unknown to him, and he also utterly ignores the use of cactin which (and also largely from Abbott's work) is now understood to be a most potent and rapidly acting cardiac tonic, the depression which might accompany the use of even pure hyoscine and morphine in full doses being prevented by the cactin present in the Abbott formula.

That this new combination permits the surgeon to perform the most bloody and tedious operations upon patients under the influence without causing pain, or the nausea and depression which follows the exhibition of ether and chloroform, matters not to Wood; nor that one busy operator has done over 300 capital operations without an untoward happening, but with the most perfect results, using the Abbott-Lanphear method.

Not one of the fatalities, which he has collected with so much pains, was caused by the "hyoscine, morphine and cactin" formula! Animosity and ignorance have led the young man to make a blunder he will find it hard to live down.

Ether, whose statistics Wood points to as almost ideal, has, it is true, maintained first place in the affections of many American surgeons, but not of all; while in Europe it has failed to dislodge chloroform. The verdict of the Hyderabad Commission placed chloroform first as to efficacy and safety, and these conclusions have never been disturbed in European surgical circles.

There has been a persistent belief that in making out such favorable statistics for ether only the immediate effects are considered—that the numerous cases of pulmonary and renal inflammation resulting fatally have been excluded. The writer has seen a patient die of total suppression of urine following ether administration, and every surgeon of experience has had his share of pulmonary edemas, broncho-pneumonias and nephrites following this anesthetic.

In other words, ether is by no means as safe an anesthetic, when remoter complications are considered, as we are often led to believe; and all things considered, there is a strong probability that when the technic of the Abbott-Lanphear hypodermic method has been carefully elaborated and its exact field of special usefulness more carefully mapped out, that it will not only equal other anesthetics in safety, but even excel them while presenting adaptations which will transcend them all.

Unfortunately few of us can maintain a judicial attitude toward methods and remedies with which we have become so familiar as to form an attachment for them. We must apply the personal equation always, and know a man's personal predilections before accurately estimating the value of his verdict. When a man is known as a special pleader for ether, we know that he is going to present the evidence for his side and suppress, or distort, or minimize, all that goes against it.

Posing as a "modern pharmacologist," as he does, Dr. Wood should familiarize himself with late discoveries and not confound scopolamine-morphine anesthesia with that product with pure hyoscine, morphine and cactin in definite proportions. In answering a question relative to the latter, he should not have inflicted upon the profession his limited knowledge of scopolamine-morphine, or of the action of pure hyoscine and morphine combined. Posing as an "authority," he should not have made the absurd statements relative to drug action which occur in his answer.

